# Hyalopathie asteroide: à propos d’une observation

**DOI:** 10.11604/pamj.2017.26.47.8601

**Published:** 2017-01-31

**Authors:** Yogolelo Asani Bienvenu, Musau Nkola Angel, Kabamba Ngombe Leon, Kapalu Mwangala Socrate, Iye Ombamba Kayimba Bruno, Chenge Borasisi Gaby

**Affiliations:** 1 Université de Lubumbashi, Faculté de Médecine, Service d’Ophtalmologie, Lubumbashi, République Démocratique du Congo; 2 Université de Lubumbashi, Faculté de Médecine, Département de Santé Publique, Lubumbashi, République Démocratique du Congo; 3 Université de Lubumbashi, Faculté de Médecine, Département de Santé Publique, Unité de Toxicologie, Lubumbashi, République Démocratique du Congo; 4 Centre Ophtalmologique Sainte Bernadette Lubumbashi, République Démocratique du Congo

**Keywords:** Hyalopathie astéroïde, diabète, vitré, Asteroid hyalopathy, diabetes, vitreous

## Abstract

Nous rapportons un cas d'hyalopathie astéroïde, rarement décrit dans la littérature, chez une personne diabétique âgée de 58 ans, de sexe masculin. Cette observation permet d'attirer l'attention des scientifiques sur les pathologies du vitré du diabétique ainsi que les autres maladies systémiques auxquelles la hyalopathie astéroïde peut être associée.

## Abstract (en)

We here report a case of a 58 year-old diabetic male patient with asteroid hyalopathy, an affection rarely described in the literature. This study can help focus the attention of scientists on the pathologies of the vitreous disorders in diabetic patients as well as on other systemic diseases asteroid hyalopathy may be associated with.

## Introduction

La hyalopathie astéroïde est une pathologie dégénérative rare du vitré [1]. Sa prévalence est estimée entre 0,15 et 0,9% dans la population [2]. Elle se rencontre surtout après 60 ans, est généralement unilatérale et est plus élevée chez les patients diabétiques [3]. Plusieurs études ont été faites sur ce sujet; elles révèlent que l'hypertension artérielle, le taux élevé des lipides sériques et le diabète sucré sont des pathologies systémiques associées. En République Démocratique du Congo, et à Lubumbashi en particulier, de telles études restent mal connues. Le but de ce travail est d'attirer l'attention des scientifiques sur les pathologies du vitré du diabétique ainsi que les pathologies systémiques auxquelles la hyalopathie astéroïde peut être associée.

## Patient et observation

**Figure 1:**
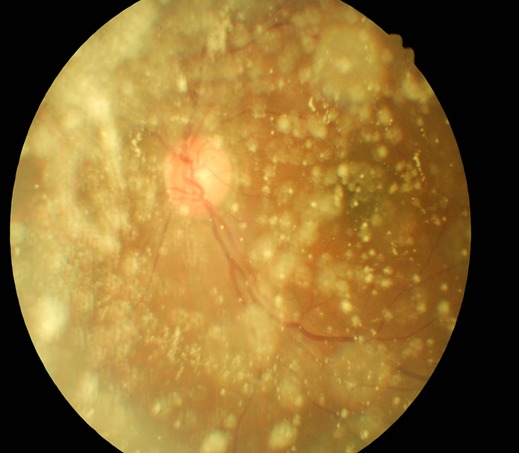
Opacités blanchâtres dans le vitré de l'œil gauche

**Figure 2:**
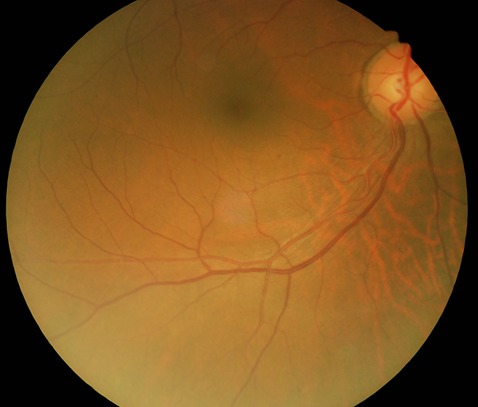
Fond d’œil normal de l'œil droit

Le patient est âgé de 58 ans; il a consulté pour douleur oculaire gauche d'environ deux semaines. Dans ses antécédents, il est diabétique connu depuis deux ans et est sous traitement. L'acuité visuelle sans correction était de 7/10 et 6/10 avec faute respectivement à l'œil droit et à l'œil gauche (Figure 1). Après correction par les verres myopiques cylindriques en inverse, l'acuité visuelle est passée à 10/10 sans faute. La tonométrie était de 18 mm Hg aux deux yeux. Le fond dilaté était normal à l'œil droit (Figure 2) et, à l'œil gauche, on notait la présence de plusieurs opacités blanchâtres, brillantes et très mobiles dans le vitré, masquant pratiquement toute la rétine et ne gênant pas la vision, faisant penser à une pathologie du gel vitréen.

## Discussion

La hyalopathie astéroïde est une affection bénigne caractérisée par de petites opacités sphériques blanches ou jaune-blanc à travers le corps vitré. L'examen biomicroscopique du vitré retrouve des opacités blanchâtres, brillantes, très mobiles avec les mouvements de l'œil. Leur nombre peut être très important, gênant considérablement l'accès à la rétine. Paradoxalement, le retentissement fonctionnel est souvent modéré avec préservation d'une bonne acuité visuelle et présence de myodésopsie peu gênante. Le vitré est généralement non décollé. La hyalopathie astéroïde peut être associée à des pathologies maculaires, et en particulier à des membranes épimaculaires, expliquant une éventuelle baisse d'acuité visuelle. Parfois, la densité des opacités est telle que l'examen de la rétine n'est plus possible, ce qui peut être problématique chez les patients diabétiques. L'angiofluorographie peut permettre un meilleur examen de la rétine chez ces patients. L'échographie en mode B montre une multitude d'éléments hyper réflectifs, très mobiles dans le gel vitréen. L'analyse histochimique prouve qu'il s'agit de dépôts phosphocalciques au niveau de la trame collagène du vitré. L'étiopathogénie est inconnue, mais son unilatéralité et sa survenue chez le diabétique font évoquer le possible rôle de facteurs vasculaires locaux [2]. Jusqu'à présent, l'étiologie de cette maladie n'est pas clairement comprise. Néanmoins, la présence de cette pathologie doit amener le chercheur a recherché le diabète chez le patient [4]. Tel est le cas avec notre observation où la hyalopathie astéroïde est présente chez un diabétique. Par contre Fawzi dans une étude rétrospective réalisée à l'université de Californie à Los Angeles a montré qu'il n'y avait pas de corrélation statistiquement significative entre hyalopathie astéroïde et diabète sucré [5, 6]. Certains auteurs ont signalé la présence de la hyalopatie astéroïde dans les deux yeux et ont conclu que le sexe masculin était le plus atteint [6, 7]. Notre observation illustre que l'atteinte est unilatérale, mais que le sexe masculin est concerné. La hyalopathie astéroïde provoque rarement des troubles visuels et l'ablation chirurgicale n'est que rarement nécessaire [8]. Notre observation l'illustre bien, quant à l'acuité visuelle qui a été améliorée par le port des verres correcteurs; aussi nous n'avons pas suggéré sa prise en charge chirurgicale. Par ailleurs, la hyalopathie astéroïde peut être associée avec certaines maladies systémiques telles que le diabète sucré, l'hypertension artérielle ou l'hyperlipidémie [9].

## Conclusion

La hyalopathie astéroïde est un diagnostic clinique et peut être secondaire ou associée à plusieurs formes de vasculopathie; le diabète sucré est une des conditions qui peuvent y être associées.
